# Composition analysis and microencapsulation of *Eucommia ulmoides* seed oil

**DOI:** 10.1186/s13065-021-00775-w

**Published:** 2021-08-23

**Authors:** Zhang Huirong, Zhang huina, Chen Nifeng, Li Li

**Affiliations:** 1 College of Chemistry and Materials Engineering, Beijing Technology and Business University, Beijing, 100048 China; 2 Beijing Key Laboratory of Plants Resource Research and Development, Beijing Technology and Business University, Beijing, 100048 China

**Keywords:** *Eucommia ulmoides* seed oil, GC–MS, Component analysis, Microencapsulation, Complex coagulation, Oxidation stability

## Abstract

**Background:**

*Eucommia ulmoides* seed oil is a functional health oil with a high content of unsaturated fatty acids. However, excessively high content of unsaturated fatty acids can cause *E. ulmoides* seed oil to easily spoil. Microencapsulation technology can effectively encapsulate substances, thereby prolonging the spoilage time of oil products.

**Methods:**

In the present study, *E. ulmoides* seed oil from different manufacturers were analyzed by Agilent 7890B-5977A gas chromatography**–**mass spectrometry. Encapsulation efficiency, yield rate, and scanning electron microscopy results between microcapsules prepared use different wall materials and different methods (spray drying and complex coagulation) were compared to determine the best preparation process for microcapsules. The Wantong 892 professional oil oxidation stability tester was used to measure the induced oxidation time of the *E. ulmoides* seed oil and microcapsules.

**Conclusion:**

*E. ulmoides* seed oil comprises > 80% unsaturated fatty acids with a high α-linolenic acid content, followed by linoleic acid. The most promising combination was chitosan:gum arabic at 1:8 as the wall material and complex coagulation. The best preparation had a wall material concentration, stirring speed, aggregation pH, and core-to-wall ratio of 2.5%, 500 rpm, 4.2, and 1:4, respectively. Microcapsules prepared under these conditions exhibited higher yield and encapsulation efficiency (94.0% and 73.3%, respectively). The induced oxidation time of the *E. ulmoides* seed oil and microcapsules were 3.8 h and 13.9 h, respectively, indicating that microencapsulation can increase the oxidation induction time of this oil.

## Introduction

*Eucommia ulmoides* Oilv. is a unique tree species in China with high economic value. It is a second-class national protected tree species in China [1] *E. ulmoides* seeds contain many active ingredients and are primarily used to produce seed oil and gum. They also contain small amounts of protein, aucubin, mineral elements, vitamins, chlorogenic acid, and crude fiber [2].

In China, *E. ulmoides* seed oil was approved as a food ingredient in the Announcement No. 12 of 2009 issued by the former Ministry of Health and the National Health and Family Planning Commission [3]. According to market surveys, using *E. ulmoides* seed oil as a raw material, there are existing products in the form of foods such as compressed candies, soft capsules, and solid beverages. *E. ulmoides* seed oil comprises a higher content of unsaturated fatty acids, including α-linolenic acid content of up to 66% [4]. It is currently considered to be one of the highest α-linolenic acid contents among vegetable oils. As an ω-3 fatty acid with three double bonds, α-linolenic acid is an essential fatty acid in the human body [5]. In addition, ω-3 polyunsaturated fatty acids play an important role in the prevention and treatment of various diseases, such as cardiovascular diseases, tumors, cancers, and some immune diseases [6], and long-term consumption has good health effects.

However, the high unsaturated fatty acid content in *E. ulmoides* seed oil makes it prone to oxidative rancidity during food processing and storage. This destroys the chemical state, sensory taste, and nutritional characteristics of the oil and produces peroxides and free radicals in the body that can lead to aging and disease. Therefore, protecting *E. ulmoides* seed oil from being oxidized is extremely important for developing its application value as a raw material [7].

Microencapsulation technology involves wrapping an active core material in a polymer to form tiny particles (microcapsules), ranging in the size of 10–1000 μm [8]. The major purpose of microcapsules is to encapsulate active substances, to ensure that the activity of the encapsulated substances is not affected by the environment in which they are used [9]. Microencapsulation is an effective method of protecting unsaturated fatty acids from oxidation.

Spray drying and complex coagulation are two widely used methods for preparing microcapsules [10]. The nature of the microcapsule wall material directly affects the stability, solubility, encapsulation efficiency, and yield of microcapsules [11]. Therefore, the choice of preparation method and wall material is extremely important.

In this study, we analyzed the ingredients of *E. ulmoides* seed oil, and selected appropriate methods and wall materials to prepared *E. ulmoides* seed oil microcapsules to attempt to improve the stability of the oil by protecting the active ingredients from light, temperature, oxygen, and other adverse factors, extending their action time. Extending the rancidity time of fats and oils has broad application prospects in food and many fields.

## Materials and methods

### Materials and instruments

*E. ulmoides* seed oils were purchased from Jiangxi Jingwang Natural Fragrance Co., Ltd., Xi'an Huageng Biotechnology Co., Ltd., Shaanxi Nexant Biotechnology Co., Ltd., and Jiangxi Hai Lin Fragrance Co., Ltd. Chitosan (CS) and gum arabic (GA) were acquired from Shanghai Macleans Biochemical Company. Hydroxyethyl cellulose (HEC) was obtained from Ashland Company. Span-80, maltodextrin (MD), sodium alginate (SA), soy protein isolate (SPI), and gelatin (Ge) were purchased from Shanghai Yuanye Biotechnology Co., Ltd. Transglutaminase was obtained from Taixing Dongsheng Food Co., Ltd.

Caprylic/capric triglyceride was purchased from Croda Cypro Chemical (Sichuan) Co., Ltd. Hydrochloric acid and sodium hydroxide (analytical grade) were purchased from Xilong Chemical Co., Ltd. Methanol (pure for mass spectrometry) was acquired from Fisher Scientific World, USA. N-hexane (analytically pure) was obtained from Shanghai Aladdin Biochemical Technology Co., Ltd. Finally, a 14% boron trifluoride methanol solution was purchased from Fuchen Chemical Reagent Co., Ltd.

### Analysis of fatty acids with GC–MS

Separately, 0.1 g of the four *E. ulmoides* seed oils were added to 1.0 mL of NaOH-CH_3_OH solution at a concentration of 0.50 mol/L, shaken well, and reacted in a 60 °C water bath for 30 min until the oil droplets disappeared completely. Then, 1 mL boron trifluoride methanol solution (0.5 mol/L) was added, and the sample was placed in a water bath at 80 °C for 5 min. The sample could cool naturally to 25 ± 1.5 °C, before 1.60 mL of n-hexane was added to the test tube, and it was shaken vigorously for extraction. The mixture could stand to clarify it, and the supernatant was collected for GC–MS analysis.

The *E. ulmoides* seed oil fatty acid methyl ester was analyzed using an Agilent Technologies 7890B-5977A gas chromatography–mass spectrometer, with an HP-5MS quartz capillary column (30 m × 0.250 mm × 0.25 μm). The column and injection port temperatures were 50 °C and 280 °C, respectively. The temperature was held at 50 °C for 2 min, increased at 5 °C/min to 180 °C, held for 5 min, and then increased at 10 °C/min to 290 °C, and held for 3 min. The carrier gas was high-purity helium (99.999%), the flow rate was 1 mL/min, the pre-column pressure was 69.8 kPa, the split ratio was 10:1, and the injection volume was 1.0 μL. Mass spectrometry conditions: electron ionization ion source 230 °C, solvent delay 3 min, electron energy 70 eV, quadrupole temperature 150 °C, scanning range 40–500 m*/z*, multiresolution signal decomposition transmission line temperature 230 °C, and electron multiplier voltage 2300 V.

The compositions of the *E. ulmoides* seed oil fatty acids were determined by comparing them with a fatty acid methyl ester mixed standard and using National Institute of Standards and Technology (NIST 14 Mass Spectral Library) MS spectrum search and matching. The relative percentage of each fatty acid methyl ester in the sample is calculated by normalizing the peak area.

### Optimal preparation of microcapsules

#### Spray drying

The wall materials listed in Table 1 were dissolved in 100.0 g of deionized water (CS was dissolved in 1% acetic acid solution). After stirring and dissolving, a certain amount of *E. ulmoides* seed oil (4.0 g) and Span-80 (2.0 g) was added. The mixture was homogenized with KINEMATICA POLYTRON® PT 2500E at 9000 rpm for 3 min to obtain an emulsion. The inlet temperature of the spray dryer (BUCHI Mini Spray Dryer B-290) was set to 130 °C, the outlet temperature was 77 °C, and the flow rate was 473 L/h. Finally, the microcapsules were collected at the sample outlet.

**Table 1:** Wall material combination of two preparation methods

Method	Wall material and proportion (A:B:C)	Ratio	Mass of wall material (g)
Spray drying	GA:MD	1:1	10.00
	CS	1	
	CS:GA	1:3	
	MD:GA:HEC	4:3:2	
	Ge:GA	1:1	
Complex coagulation	GA:CS	8:1	5.63
	Ge:GA	1:2	
	Ge:CS	8:1	
	Ge:HEC	4:1	
	Ge:SA	8:1	
	SPI:CS	6:1	

#### Selection of wall material ratio in complex coagulation method

The turbidity method was used to select the optimal ratio of the six wall combinations for the complex coagulation method in Table 1. The required wall solutions in different ratios (8:1, 6:1, 4:1, 2:1, 1:1, 1:2, 1:4) were configured separately and one wall solution was added to the other wall solution. After the final mixing, the total concentration of the wall material solution was 2.0% (*w/w*), the pH was adjusted to 4.0, and the absorbance was measured at 600 nm using a microplate reader (Dicken (Shanghai) Trading Co., LTD Infinite M200 Pro). The turbidity liquid was poured into a centrifuge tube and mixed at 5 000 rpm (25 °C) for 10 min, and the absorbance of the supernatant was measured.

#### Complex coagulation

The wall-to-material ratio determined by the turbidity method is shown in Table 1. Core material A of the mass shown in Table 1 was weighed and dissolved in 50.0 g of deionized water, heated to 45 °C, and stirred until fully dissolved. Then, 4.0 g of *E. ulmoides* seed oil and 2.0 g Span-80 were added, and the mixture emulsified at 8000 rpm for 3 min. Core material B was dissolved in 50.0 g of deionized water for later use. The emulsified mixed solution was stirred at 300 rpm and slowly drip into the dissolved material B solution, which was then stirred for 10 min. The pH of the stirred solution was adjusted to 4.0, continue to stir for half an hour and the solution was placed in a 5 °C ice bath for 30 min. After the ice bath, the pH was adjusted to 6.5, 0.50 g of transglutaminase was added, and the temperature was slowly raised to 30 °C. The mixture was stirred for 30 min, and centrifuged, then washed precipitation (microcapsules). The microcapsules were freeze-dried and stored.

### Determination of microencapsulation efficiency and yield

#### Encapsulation efficiency

The encapsulation efficiency (EE, %) was assessed in two steps. For the surface oil measurement, microcapsules (0.20 g) were dissolved in 5.0 mL n-hexane and centrifuged at 7000 rpm for 5 min. The supernatant was collected, the absorbance was measured at the maximum wavelength, and the standard curve (established at the maximum wavelength of the *E. ulmoides* seed oil.) was used to calculate the concentration of *E. ulmoides* seed oil. For the total oil measurement, microcapsules (0.20 g) were dissolved in 5.00 mL n-hexane in a shaker for 12 h and sonicated for 1 h. The solution was centrifuged at 7000 rpm for 20 min, the supernatant was collected to measure its absorbance at the maximum wavelength, and the standard curve was used to calculate the concentration. EE was calculated as follows:

$$ EE\left( \% \right) = \left( {1 - SO/ TO} \right) \times 100, $$

where *TO* and *SO* denote the total oil and surface oil contents, respectively.

#### Microcapsule yield

The microcapsule yield was calculated as follows:

$$ Y = M/\left( {m_{AB} + m} \right), $$

where *M, m*_*AB*_, and *m* represent the sample quality, total mass of wall materials A and B, and mass of the core material, respectively.

### Scanning electron microscopy

A scanning electron microscope (JEOL JSM-6700) was used to observe the morphology of the *E. ulmoides* seed oil microcapsules and determine the combination of wall materials formed by complex coagulation. The microcapsules prepared by complex coagulation were evenly scattered across the sampling table of the scanning electron microscope, and any excess, unadhered sample were blown off. The morphology and structure of the microcapsules were observed and photographed at appropriate positions and magnifications. The acceleration voltage of the scanning electron microscope was 10.0 kV.

2.6 Optimization of the preparation process using complex coagulationThe effects of the wall material concentration (1.5, 2.0, 2.5, 3.0, 3.5, and 4.0%), stirring speed (100, 300, 500, 700, and 900 rpm), aggregation pH (3.8, 3.9, 4.0, 4.1, and 4.2), and core-wall ratio (1:4, 1:3, 1:2, 1:1, and 2:1) on the microcapsules were studied.

### Microcapsule morphological observation and particle size measurement

The morphology of each microcapsule sample was observed using a polarizing microscope. The microcapsule turbid solution (0.1 mL) was diluted with excess water. The diluted microcapsules were placed on a glass slide, and the appropriate light source and magnification were selected for observation and photography.

The median particle size of each microcapsule suspension was measured using a Malvern Mastersizer 3000 laser diffraction particle size analyzer. The median droplet diameter of the *E. ulmoides* seed oil emulsion was measured after emulsification at 10,000 rpm for 3 min. The aggregation index of the microcapsules, according to the median sizes of the emulsion and microcapsule suspension particles, was calculated as follows:

$$ In = \left( {S_{m} - S_{e} } \right) / S_{e} $$

where *I*n is the aggregation index, *S*_*m*_ the microcapsule size, and *S*_*e*_ is the emulsion particle size.

### Oxidation induction

The method used for determining the oxidation induction time was in accordance with the methods specified in GB/T 21121–2007/ISO6886:2006. The Wantong 892 professional oil oxidation stability tester was used to determine the oxidation induction time of the *E. ulmoides* seed oil and microcapsules. A sample (0.60 g) of microcapsule powder or *E. ulmoides* seed oil was placed into a test tube. The accelerated oxidation temperature was set to 120 °C, and the air flux to 20 L/h. Then, 60.0 g of purified water was received from the pool, and the oxidation induction time was recorded.

### Data analysis

All data is based on at least three parallel experiments, and the results are expressed as the mean ± SD. Excel and Origin software were used to process the data, and Duncan’s test was used to analyze the significance of differences between multiple groups of samples. The significance level was set to 95% (*p* < 0.05).

## Results and discussion

### GC–MS for fatty acid component analysis

**Fig. 1:**
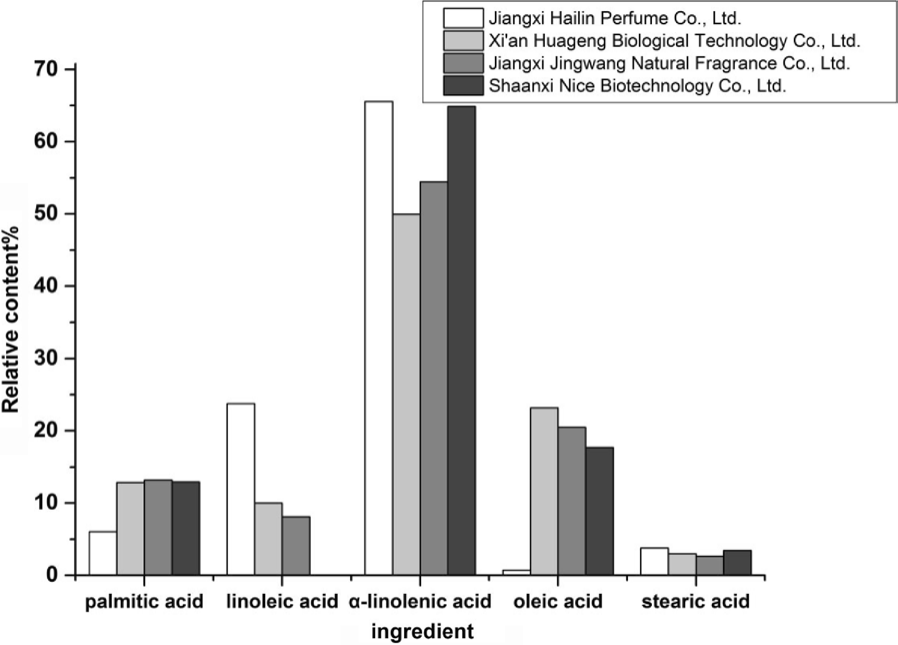
Composition and relative content of mixed fatty acid of different batches of *E. ulmoides* seed oil

The GC–MS chromatograms of the four *E. ulmoides* seed oils have extracted 13–15 chromatographic peaks, ignoring the components whose content is less than 0.5%. Under these conditions, the composition and relative content of the mixed fatty acid methyl esters of the *E. ulmoides* seed oils are presented in Fig. 1. According to our analysis, the ingredients of the four *E. ulmoides* seed oils are similar. They all contain α-linoleic acid, palmitic acid, linoleic acid, oleic acid, stearic acid and other relatively small (< 0.5%) amounts of ingredients (such as arachidic acid, etc.). Among them, the unsaturated fatty acids are mainly α-linoleic acid (50–65%), linoleic acid (5–25%), oleic acid (< 25%), and the total amount > 80%. Based on comprehensive considerations, this paper finally chose *E. ulmoides* seed oil from Jiangxi Jingwang Natural Fragrance Co., Ltd. for follow-up experiments.

### Determination of wall material ratio

The turbidity method was used to compare the aggregation effects of different ratios of wall material combinations. The difference between the optical density of the composite turbid liquid and the optical density of the supernatant after centrifugation was used as an index. The optimal ratios of wall material combinations were obtained when the pH was 4.0 and the wall material concentration was 2.0% (*w/w*). They were CS:GA = 1:8, Ge:GA = 1:2, CS:Ge = 1:8, HEC:Ge = 1:4, SA:Ge = 1:8, and CS:SPI = 1:6.

### Encapsulation rate and yield of microcapsules

#### Encapsulation rate and yield of the spray drying formula

**Fig. 2:**
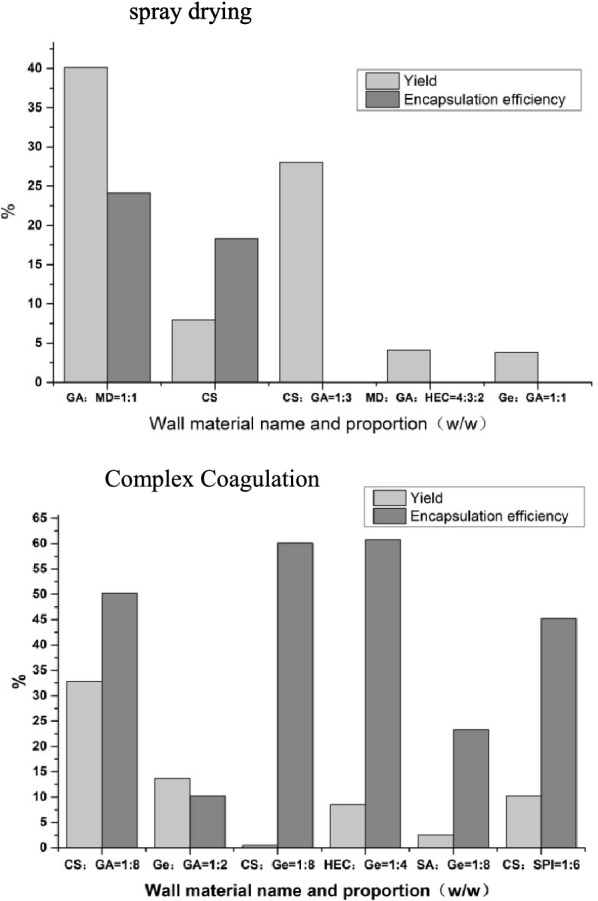
The yield and encapsulation efficiency of different formulations of spray drying and complex coagulation

The EEs and yields of five formulations produced using the spray drying method were obtained (Fig. 2). The microcapsules prepared using formula 1 displayed a yield of 40.1% and an EE of 24.1%, which were the highest values among the five formulas. This shows that the seed oil was not effectively encapsulated and could not be formed into microcapsules.

#### Encapsulation rate and yield of the compound coagulation method

The yields and EEs of three parallel experiments were obtained (Fig. 2). Using GA and CS as the wall materials to prepare the *E. ulmoides* seed oil microcapsules produced the highest yield and EE (32.8% and 50.2%, respectively). The yields of the other wall material combinations did not exceed 15.0%. HEC and Ge will produce higher viscosity at small concentrations; Ga and CS will undergo agglomeration reaction at room temperature, which is not conducive to sample injection, so it is difficult to effectively prepare microcapsules.

#### Choice of preparation method

A comparison of these two methods revealed that, although spray drying uses simple operating equipment, the yield and EE of the resultant microcapsules were poor. The complex coagulation method also has a low cost and simple operation. The method could effectively coagulate and precipitate the samples to form microcapsules, under specific conditions of pH adjustment and cooling. When comparing the microcapsule yields and EEs of the two preparation methods, the complex coagulation method fared better than the spray drying method. Consequently, complex coagulation was selected as the preparation method for the microcapsules.

### Selection of wall materials

**Fig. 3:**
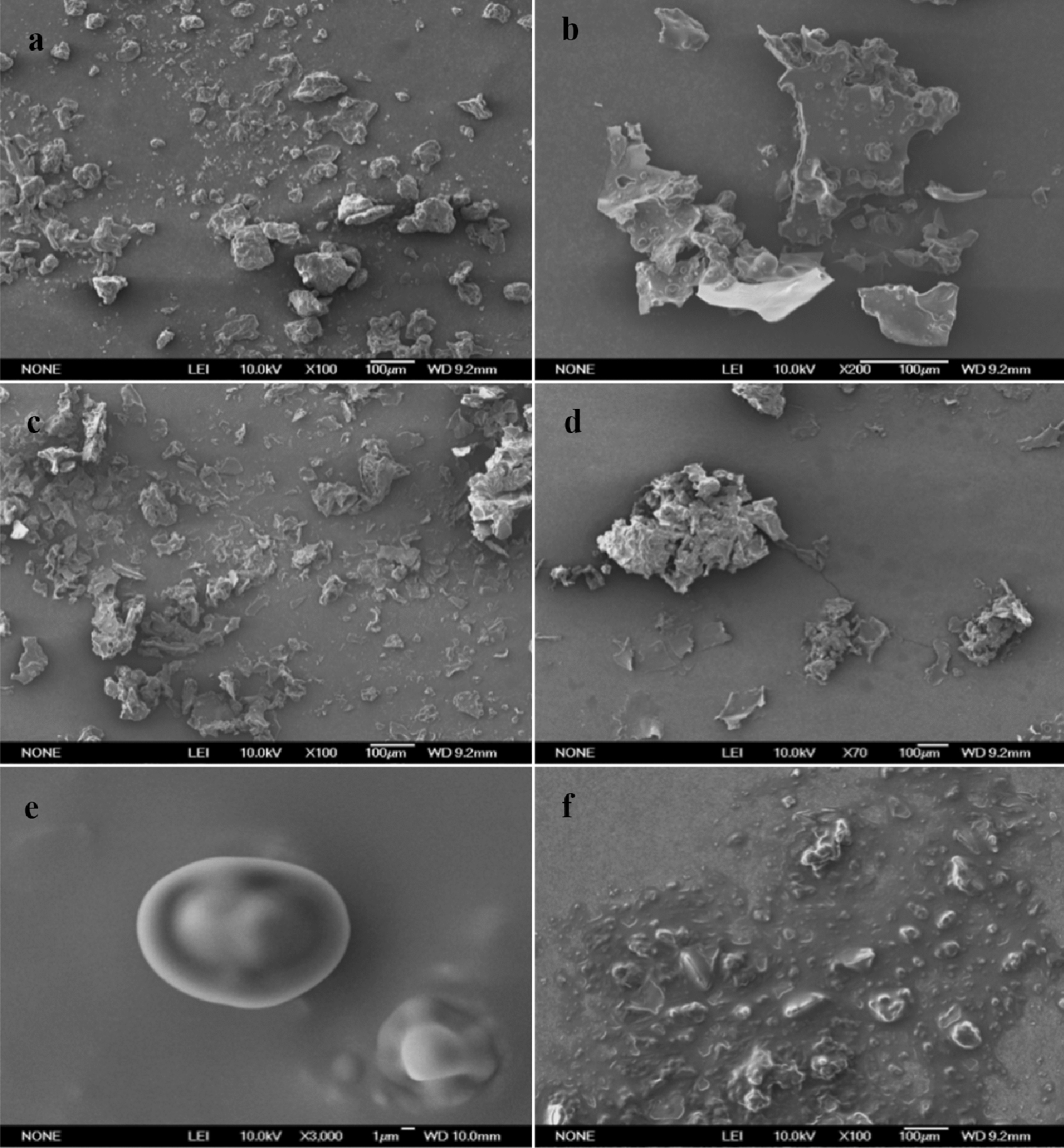
SEM images of different wall material combinations of *E.ulmoides* seed oil microcapsules prepared by complex coagulation method: **a** GA:CS **b** Ge:GA **c** Ge:CS; **d** Ge:HEC; **e** Ge:SA; **f** SPI:CS

The 6 kinds of microcapsule samples with different wall materials prepared by the complex coagulation were observed by scanning electron microscope, and the final choice of wall material combination is determined by the shape of the microcapsules. In the images (Fig. 3), the microcapsules prepared with GA and CS as the wall materials exhibited a more uniform particle shape. The microcapsules prepared from other wall materials were either irregular flakes, instead of spherical microcapsules (Fig. 3b, c, d), or if spherical, showed oil leakage on the surface (Fig. 3e), or may adhere to each other and have irregular shapes (Fig. 3f). After considering the EEs and yields, GA and CS (Fig. 3a) were selected as the most appropriate wall materials for the microcapsules.

### Encapsulation efficiency and yield of process optimization

Various *E. ulmoides* seed oil microcapsules were prepared using a single factor method to determine the best preparation process. Four factors (concentration, stirring speed, aggregation pH, and core-wall ratio) were investigated. The EEs and yields of the microcapsules were used as indicators.

#### Wall material concentration

**Fig. 4:**
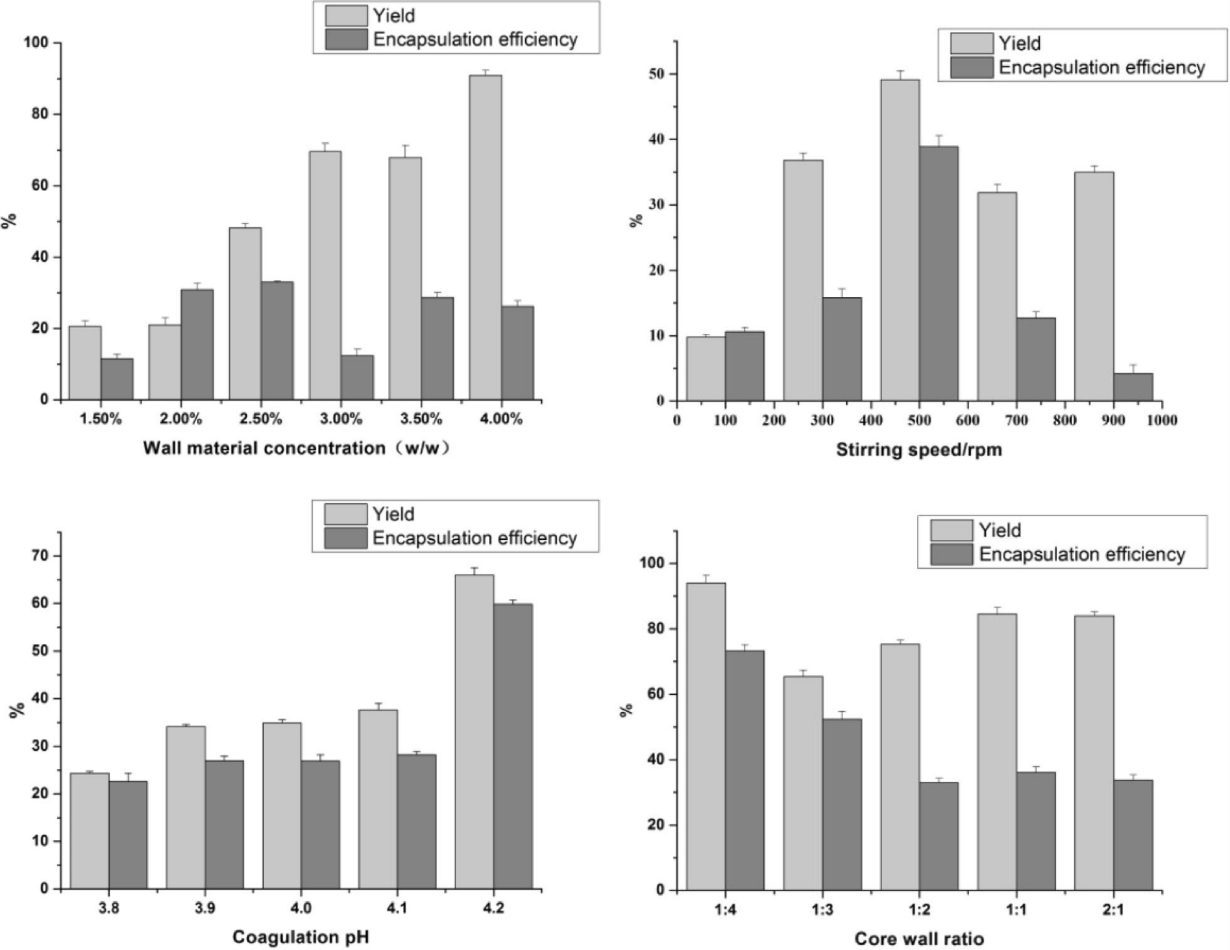
The yield and encapsulation efficiency of microcapsules under different processes

The stirring speed was 400 rpm, the aggregation pH was 4.0, the core-to-wall ratio was 5.63:4.00 (*w/w*), and the wall material concentration was changed (Fig. 4). With the increasing wall material concentration, the yield of the microcapsules continued to increase, but the EE and yield trends were different. At a concentration of 2.5% (*w/w*), the highest EE was reached (33.0%), and the yield was also acceptable (48.1%). At higher concentrations, the viscosity of the solution increased, which was not conducive to microcapsule preparation.

#### Stirring speed

The core-to-wall ratio was 5.63:4.00 (*w/w*), the agglomeration pH was 4.0, the wall material concentration was 2.5%, and the stirring speed was changed (Fig. 4). As the rotation speed increased, the EE and yield of the microcapsules showed a trend of first increasing and then decreasing. At a low stirring speed, the wall and core materials could not be uniformly dispersed to form microcapsules. An extremely high stirring speed was also not conducive to microcapsule formation. The yield and EE of the microcapsules both reached a maximum at 500 rpm.

#### Coagulation pH

The core-to-wall ratio was 5.63:4.00 (*w/w*), the stirring speed was 500 rpm, the wall material concentration was 2.5%, and the agglomeration pH was changed (Fig. 4). With increasing aggregation pH, the yield and EE of the *E. ulmoides* seed oil microcapsules continued to increase, and they were both highest at a pH of 4.2.

#### Core-to-wall ratio

The stirring speed was 500 rpm, the agglomeration pH was 4.2, the wall material concentration was 2.5%, and the core-to-wall ratio was changed (Fig. 4). With an increasing core-to-wall ratio, the yield and EE of the microcapsules continued to decrease. When the core-to-wall ratio was 1:4 (*w/w*), the yield and EE both reached their highest values.

#### Optimal process conditions

The optimization of the microcapsule preparation process greatly improves the EE of the microcapsules. The concentration of 2.5% (*w/w*), stirring speed of 500 rpm, pH of 4.2, and core-to-wall ratio of 1:4 (*w/w*) were preliminarily determined the best process conditions for agglomeration. Under these conditions, the highest yield of the microcapsules was 94.0%, and the EE reached 73.3%.

### Influence of preparation processes on microcapsule morphology

The preparation of microcapsules by complex coagulation will produce mononuclear and multinuclear microcapsules. The larger the aggregation index, the lower the degree of mononuclear microcapsules, and vice versa. When preparing microcapsules using the complex coagulation, any change in the preparation process will have a significant impact on the morphology and particle size of the final microcapsules. Therefore, the influence of different conditions on the morphology and particle size of the microcapsules is discussed below.

#### Wall material concentration

**Fig. 5:**
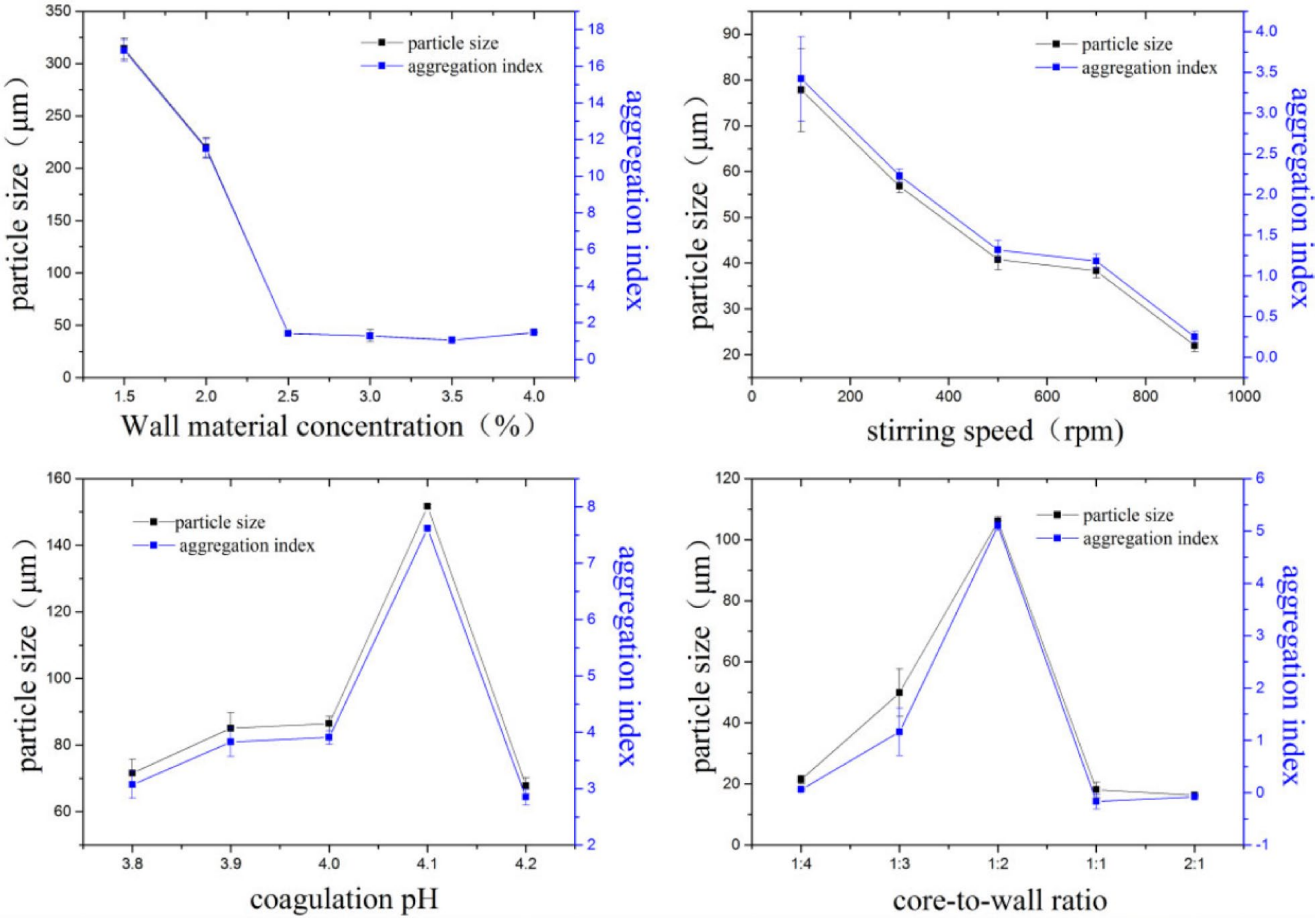
Particle size and aggregation index of microcapsules under different conditions

The influence of wall material concentration, in the range of 1.0–4.0% (*w/w*), on the morphology and structure of the microcapsules was explored (Fig. 5). As the wall material concentration increased, the microcapsule particle size decreased. When the wall material concentration was 1.5–2.0% (*w/w*), the particle size and aggregation index of the microcapsules were too large. At wall material concentrations in the range of 2.5–3.5% (*w/w*), the aggregation index reached its lowest level, and the microcapsules were all mononuclear. Thus, 2.5–3.5% (*w/w*) can be initially chosen as the wall material concentration for mononuclear microcapsules.

#### Stirring speed

With increasing stirring speed, the particle size of the *E. ulmoides* seed oil microcapsules decreased (Fig. 5). At stirring speeds of 100–300 rpm, the particle size and aggregation index of the microcapsules were too large. The polarized light microscope observations also showed that these microcapsules were multi-cored. In the range of 500–900 rpm, the aggregation index reached its lowest level, and the microcapsules were all single-core structures. When the stirring speed was further increased to 900 rpm, the aggregation index was slightly lower, and the encapsulation effect was poor. Thus, 500–700 rpm can be initially chosen as the stirring speed for preparing the microcapsules.

#### Coagulation pH

The influence of the coagulation pH, in the range of 3.8–4.2, on the morphology and structure of the microcapsules was explored (Fig. 5). As the pH increased, the microcapsule aggregation index first increased and then decreased. At a pH of 4.2, the microcapsule particle size was the smallest with appropriate dispersion. Therefore, pH of 3,8 and 4.2 can be initially chosen for preparing the microcapsules.

#### Core-to-wall ratio

As the core-to-wall ratio increased, the microcapsule aggregation index first increased and then decreased (Fig. 5). When the core-to-wall ratios were 1:1 (*w/w*) and 2:1 (*w/w*), the aggregation index was negative, and it was difficult to embed the oil droplets. When the core-to-wall ratio was 1:2 (*w/w*), the aggregation index was at its highest. Thus, 1:4 and 1:3(*w/w*) can be initially chosen as the core-to-wall ratio.

#### Optimal process conditions

It can be found from Fig. 5 that the change trend of the aggregation index is similar to that of the microcapsule particle size. Comprehensive consideration of EE, yield and microcapsule morphology, and finally determine the best preparation process of microcapsules (concentration of 2.5%, stirring speed of 500 rpm, pH of 4.2, and core-to-wall ratio of 1:4). The microcapsules prepared under this process have high EE and yield, and meanwhile, have good morphology and uniform particle size(47.6 ± 5.37 μm).

### Microcapsule oxidation induction time

An oil oxidation stability tester was used to determine the oxidation induction time of the *E. ulmoides* seed oil and microcapsules. At an accelerated oxidation temperature of 120 °C and an aeration rate of 20 L/h, the oxidation induction time of the *E. ulmoides* seed oil was only 3.8 h. After microencapsulation, the oxidation induction time of the oil was extended to 13.9 h.

This shows that the wall material acts as a physical barrier and has a protective effect on the core material. Therefore, the oxidation induction time, stability, and survival period of *E. ulmoides* seed oil can be increased by microencapsulation.

## Conclusion

In this study, GC–MS was used to analyze the composition of *E. ulmoides* seed oil. After screening potential wall materials and optimizing the process, a complex coagulation was used to prepare microcapsules with higher encapsulation efficiency, good morphology and stability. The oxidation stability of the microencapsulated *E. ulmoides* seed oil was also studied. The specific results are as follows:

- The GC–MS analysis of the *E. ulmoides* seed oil showed that *E. ulmoides* seed oil primarily comprises stearic acid, palmitic acid, palmitoleic acid, oleic acid, linoleic acid, and α-linolenic acid. The unsaturated fatty acid content was relatively high (up to 80%), and the α-linolenic acid content was 50–65%.
- The complex coagulation method was selected, and microcapsules were prepared with CS and GA as wall materials. Then, the optimal preparation conditions of the complex coagulation method were determined. The optimal ratio of CS to GA was 1:8 (*w/w*), the wall material concentration was 2.5% (*w/w*), the stirring speed was 500 rpm, the pH was 4.20, and the core-to-wall ratio was 1:4 (*w/w*). Under these optimal preparation conditions, the highest yield of the microcapsules reached 94.0%, and the EE reached 73.3%. The microcapsules are spherical and uniform in size (47.6 ± 5.37 μm).
- After microencapsulation, the complete oxidation induction time of seed oil was extended from 3.8 to 13.9 h. This shows that the oxidative stability of *E. ulmoides* seed oil is significantly improved after encapsulation using microcapsules.

The process route is simple and practical, and the realization of microencapsulation has strong operability, and can be extended to the encapsulation of other oils.. The microencapsulation of *E. ulmoides* seed oil improves the storage shelf life, and effectively solves the problems of seed oil being prone to rancidity and deterioration in storage stability. Moreover, the microencapsulated products are more convenient to be added to foods as food additives for the development of functional foods, which broadens the application fields of the *E. ulmoides* seed oil.

## Data Availability

The data used to support the fndings of this study are available from the cor-responding author upon request.

## Abbreviations

E*. ulmoides* seed oil: *Eucommia ulmoides* Seed oil
GC–MS: Gas chromatography**–**mass spectrometry
CS: Chitosan
GA: Gum Arabic
HEC: Hydroxyethyl cellulose
MD: Maltodextrin
SA: Sodium alginate
SPI: Soy protein isolate
Ge: Gelatin
EE: Encapsulation efficiency
